# Editorial: The phenomenon of misinformation in different domains and by various disciplines

**DOI:** 10.3389/fpsyg.2025.1763023

**Published:** 2026-01-13

**Authors:** Marco Biella, Veronika Batzdorfer

**Affiliations:** 1 Faculty of Business and Economics, University of Basel, Basel, Switzerland; 2 Department of Sociology and Computational Sociology, Karlsruhe Institute of Technology, Karlsruhe, Germany

**Keywords:** AI, fake news, misinformation, social media, sociology, trust erosion

This editorial has two main goals: first, to introduce the manuscripts that comprise the Research Topic (see Research Topic), and second, to offer provocative suggestions intended to stimulate the debate on misinformation research (see Outstanding Questions). After reading the editorial, readers will have all they need to navigate the papers, and they will be prompted to form a new and original take on misinformation.

## Research Topic

The Research Topic contains manuscripts addressing misinformation from a broader perspective. At the same time, this Research Topic targets social media or social media formats. This common perspective is warranted as these channels offer great opportunities and interesting paradoxes in the context of misinformation research. For example, social media are valuable tools to tackle/track conspiracy theories' spreading dynamics in the context of worldwide societal issues such as the COVID-19 pandemic (Batzdorfer et al., 2022). However, the use of these channels seems to be a protective factor against related and societally relevant issues such as vaccination hesitancy (Biella et al., 2023). This Research Topic chases this avenue and tentatively offers novel insights on the larger picture framing misinformation and its consequences at the societal level. To begin with, the first manuscript focuses on trust toward others (Zhou et al.), suggesting mechanisms triggering trust erosion and potential coping strategies. Although this manuscript does not target misinformation directly, it touches upon constructs such as trust in others and social cohesion, which are an inevitable byproduct of the discourses in which responders are embedded. This placement alone warrants the inclusion of the manuscript in the larger landscape of media and misinformation research. Another manuscript investigates trust in government and the role of Deepfakes in shaping the perception of government bodies (Ahmed et al.). Focusing on visual media, Deepfakes have the potential to exert unprecedented persuasion on users consuming its products. Users exposed to fake information in the form of AI-generated pictures cannot inhibit their attribution processes, which inevitably taints the perception of the presumed perpetrator. A third manuscript explicitly addresses cognitive and emotional processes in neglected populations, such as adolescents (Wang et al.). Adolescents are known to be social media over-users who particularly respond to emotionally appealing content. Their reactions to such content are mostly due to their ongoing emotional development. Notably, the manuscript advances an easy and effective coping strategy to ameliorate the potential harm of these contents. Finally, the fourth manuscript highlights that truth judgment extends beyond trust (Bertram et al.). It challenges the widespread belief that trust propensity or social trust might facilitate falling for fake news. The manuscript provides evidence against such a belief, calling on researchers to identify plausible precursors of truth judgments. Moreover, the investigation calls for the need to reassess the implicit hypothesis in misinformation and trust research, which suggests that individual differences are somewhat related to susceptibility to misinformation or truth biases.

From these four manuscripts, we can extract the current state of misinformation research focusing on the digital realm, unpacking why people share information online, spreading true and fake news alike, and how misinformation threatens the very fabric of human society. At the same time, these manuscripts advance promising coping strategies, which are mostly emotional in their points of attack (Wang et al.) or based on social cohesion (Zhou et al.). Similarly, they highlight potential pitfalls that the field might encounter (Bertram et al.).

## Outstanding questions

We hope the readers will go through the Research Topic with a few questions at the back of their mind, which might trigger the critical development of new stances and research avenues. For example, the reader might notice the individualistic perspective that permeates misinformation research, including this Research Topic. Most published literature in this space, as well as in the whole of psychological research, takes single respondents as units of analysis, almost neglecting the larger nature of the debate on misinformation. This phenomenon can also be investigated with a wider societal perspective, involving long-term historical processes, critically engaging with the goals of many (individual or institutional) news producers, or adopting a sociological perspective. Any pieces of misinformation must originate as information of some kind in the first place. As such, it must be produced by someone to serve a goal that could be anything ranging from personal entertainment to deliberate propaganda. On top of that, the content producer might either be genuinely convinced of the content's veracity or aware of its falsehood. This last feature, in combination with the problematic nature of the goal, will inform tools used to conduct the investigation and the conclusions that can be drawn. In short, misinformation can be framed as a special case of what Durkheim referred to as *collective representations*, but putting such a representation in context requires adopting a larger sociological perspective. Both the sociological and the traditional individualist perspectives are worth pursuing, but picking one over the other should be a deliberate decision of the researcher. To counteract the current imbalance overrepresenting research endorsing a more individualistic view over research endorsing a sociological perspective, we would like to present the words used by Wagner (2011) to describe social representation theory.

“*The theory has proved useful in research that transcends the traditional individualist assumptions of social psychology and targets societal problems in the fields of social conflict, popularized science, and the cultural dynamics of modern societies*.“ (Wagner, 2011, p. 5).

We would like to promote these words from a simple observation on the use of a specific theory to an open call addressed to all misinformation researchers. We hope that pointing researchers' attention to this tool, which we believe is underused in the context of misinformation research, might spawn interesting research avenues and theoretical ideas.

Concluding this editorial, we would like to remark that our Research Topic offers interesting suggestions for further research, but it is inevitably limited to a partial Research Topic of investigations. The field can only advance through further investigations that foster the debate on a societally relevant issue, which must remain under the spotlight.
